# Non-invasive mechanical ventilation in patients with diffuse interstitial lung diseases

**DOI:** 10.1186/1471-2466-14-194

**Published:** 2014-12-05

**Authors:** Stefano Aliberti, Grazia Messinesi, Silvia Gamberini, Sveva Maggiolini, Dina Visca, Vanni Galavotti, Fabio Giuliani, Roberto Cosentini, Anna Maria Brambilla, Francesco Blasi, Raffaele Scala, Mauro Carone, Francesca Luisi, Sergio Harari, Antonio Voza, Antonio Esquinas, Alberto Pesci

**Affiliations:** Department of Health Science, Clinica Pneumologica, AO San Gerardo, University of Milan Bicocca, Via Pergolesi 33, Monza, Italy; SC di Pneumologia e UTIR, Azienda Ospedaliera C. Poma, Mantova, Italy; Interstial Lung Disease Unit, Royal Brompton Hospital, Sydney St, London, UK; Department of Clinical and Experimental Medicine, Respiratory Disease Unit, University of Parma, Parma, Italy; Dipartimento di Fisiopatologia e dei Trapianti, University of Milan, IRCCS Fondazione Ca’ Granda Ospedale Maggiore Policlinico, Via F. Sforza 35, Milan, Italy; Emergency Medicine Department, IRCCS Fondazione Ca’ Granda, Ospedale Maggiore Policlinico, Via F. Sforza 35, Milan, Italy; Pulmonary Unit and Respiratory Intensive Care Unit, Ospedale S. Donato, Via P. Nenni, 20, Arezzo, Italy; Divisione di Pneumologia, IRCCS Fondazione Salvatore Maugeri, Istituto Scientifico di Cassano delle Murge, Bari, Italy; U.O. di Pneumologia e Terapia Semi-Intensiva Respiratoria, Servizio di Fisiopatologia Respiratoria ed Emodinamica Polmonare, Ospedale San Giuseppe - MultiMedica, Milan, Italy; Emergency Medicine Department, Humanitas Research Hospital, Via Manzoni 56, Rozzano Milan, Italy; Intensive Care Unit, Hospital Morales Meseguer, Murcia, Spain

**Keywords:** Fibrosis, Diffuse parenchymal lung disease, Non-invasive ventilation, Interstitial lung disease, Pneumonia, Continuous positive airway pressure, Ventilation

## Abstract

**Background:**

To evaluate noninvasive ventilation (NIV) in diffuse interstitial lung diseases (DILD) patients with acute respiratory failure (ARF) according to baseline radiological patterns and the etiology of ARF.

**Methods:**

In a multicenter, observational, retrospective study, consecutive DILD patients undergoing NIV because of an episode of ARF were evaluated in six Italian high dependency units. Three groups of patients were identified based on the etiology of ARF: those with pneumonia (Group A), those with acute exacerbation of fibrosis, (Group B) and those with other triggers (Group C). Clinical failure was defined as any among in-hospital mortality, endotracheal intubation and extra-corporeal membrane oxygenation use.

**Results:**

Among the 60 patients enrolled (63% males; median age: 71 years), pneumonia (42%) and acute exacerbation of fibrosis (39%) were the two most frequent causes of ARF. A significant increase of PaO_2_/FiO_2_ ratio during NIV treatment was detected in Group A (p = 0.010), but not in Group B. No significant difference in PaO_2_/FiO_2_ ratio, PaCO_2_ and pH values during NIV treatment was detected in patients with a radiological pattern of usual interstitial pneumonia (UIP) and non-specific interstitial pneumonia (NSIP). 22 patients (37%) suffered for a clinical failure. No significant differences in the study outcome were detected in Group A vs. Group B, as well as among patients with a radiological pattern of UIP vs. NSIP.

**Conclusions:**

NIV treatment should be individualized in DILD patients with ARF according to the etiology, but not the baseline radiological pattern, in order to improve oxygenation.

## Background

Various diffuse interstitial lung diseases (DILD) have different etiologies and heterogeneous radiological patterns [1, 2]. The natural history of DILD is characterized by life-threatening episodes of acute respiratory failure (ARF) triggered by known causes, such as pulmonary infections and acute heart failure. When an acute deterioration is of unknown etiology, it is termed acute exacerbation of pulmonary fibrosis [3].

During an episode of ARF, mechanical ventilation may be considered a therapeutic option in patients with DILD. Recruitment of poorly ventilated alveoli, unloading of respiratory muscles, favorable hemodynamic impact on coexisting decompensated acute heart failure, constitute the potential patho-physiological rationale for the use of mechanical ventilation during ARF in these patients. However, clinical benefits offered using this ventilatory strategy are not well documented, and admission to ICU and invasive mechanical ventilation are associated with poor outcomes in patients with DILD [4, 5].

Noninvasive ventilation (NIV) has been recognized as a means to avoid intubation during ARF and to reduce the risk of complications, such as ventilation-associated pneumonia, especially in immunosuppressed patients [6]. NIV could be a valuable option for management of respiratory failure in patients with DILD, especially if an early treatment is initiated [7–9]. Recent literature has shown that the survival of DILD patients receiving NIV seems to be higher in comparison to those who require invasive mechanical ventilation [4].

As well as the spectrum of DILD being extremely heterogeneous, the response to NIV may vary from patient to patient. The efficacy of NIV in these patients during ARF may depend on two factors. From one hand, the application of a positive pressure could lead to different results according to the natural history of the DILD that is worse in the presence of usual interstitial pneumonia (UIP) compared to non-specific interstitial pneumonia (NSIP) and other radiological and pathological patterns. From the other hand, the efficacy of NIV treatment could strictly depend on the etiology of the ARF whether a potentially reversible trigger (i.e.: pneumonia/acute heart failure) or an acute exacerbation of pulmonary fibrosis occurs.

The aim of this study was to evaluate the efficacy of NIV on gas exchange improvement and clinical outcomes in patients with DILD undergoing an episode of ARF, according to baseline radiological patterns and etiology of the ARF.

## Methods

### Study design and participants

This was a multicenter, observational, retrospective study of consecutive patients with DILD undergoing NIV due to an episode of ARF at six Italian high dependency units (HDU) between January 2004 and December 2009. The institutional review board of the San Gerardo Hospital, Monza, Italy, approved the study, and the informed consent was waived owing to the retrospective nature of the study.

Records of all the enrolled patients were carefully reviewed. Data on admission and during NIV treatment were collected and included the following: a) demographic information and past medical history; b) clinical, laboratory and radiological characteristics; c) clinical outcomes including endotracheal intubation (ETI), treatment with extra-corporeal membrane oxygenation (ECMO) and in-hospital mortality. All data were electronically sent to the San Gerardo Hospital, Monza, Italy. A group of investigators at the HDU of the San Gerardo Hospital validated data quality by checking for discrepancies and inconsistencies before cases were entered into a database. Institutional review board approval was waived in view of the retrospective design of the study.

Each case, along with radiological findings, was presented to a clinical review committee to confirm the presence and the type of DILD before hospitalization, including a UIP and NSIP pattern. All available clinical, functional and pathological data from bronchoalveolar lavage and lung biopsy were carefully evaluated in each patient. The review committee also defined the etiology of the ARF. The review committee was composed of five pulmonary and critical care physicians (SA, GM, SG, FG and AP). All reviewers had clinical and research experience on both pulmonary fibrosis and non-invasive ventilation.

NIV was administered as non-invasive pressure support ventilation (PSV) with a high-performance ventilator, including Evita 4 (Drager), VELA (Care Fusion), Servo 300 (Maquet) and Esprit (Philips Respironics), or high-flow stand-alone non-invasive continuous positive airway pressure (CPAP). Criteria for application of CPAP in the study centers included the presence of both severe acute respiratory failure (PaO_2_/FiO_2_ ratio less than 200) and respiratory rate exceeding 30 breaths/minute or use of accessory respiratory muscles or paradoxical abdominal motion, in the absence of respiratory acidosis (pH < 7.35, PaCO_2_ ≥ 45 mmHg). Criteria for application of PSV in the study centers included the presence of respiratory acidosis (pH < 7.35, PaCO_2_ ≥ 45 mmHg) and a respiratory rate exceeding 30 breaths/minute or use of accessory respiratory muscles or paradoxical abdominal motion. NIV was not applied if any of the following was present: 1) immediate need for endotracheal intubation; 2) severely altered consciousness (Kelly score > 3); and 3) shock despite fluid optimization and use of vasopressor. Medical treatment was performed according to the trigger of ARF and local standard procedures. No subjects receiving invasive or non-invasive pressure support ventilation before PSV/CPAP treatment were included in this study.

### Study definitions

A UIP pattern on high resolution CT (HRCT) scan of the thorax was defined by the presence of basal-predominant reticular abnormality, mainly peripheral and subpleural, characterized by honeycombing with or without traction bronchiectasis/bronchiolectasis. A NSIP pattern on thorax HRCT was defined by the presence of peripheral, subpleural, basal ground glass attenuation and reticular opacity with or without consolidation, as previously described [1].

Pneumonia was defined as the presence of a new pulmonary infiltrate on chest radiograph or CT scan at the time of hospitalization associated with one or more of the following: (1) new or increased cough with or without sputum production; (2) fever (> = 37.8°C) or hypothermia (<35.6°C); or (3) abnormal white blood cell count (either leukocytosis or leukopenia), or C-reactive protein values above the local upper limit.

Acute exacerbation was defined as an acute, clinically significant deterioration of unidentifiable cause in patients with underlying NSIP or UIP including: 1) worsening of dyspnea within days to weeks (<30 days); 2) evidence of a worsening of gas exchange; 3) new radiographic opacities; and 4) an absence of an alternative explanation, such as infection, left heart failure, pulmonary embolism or pneumothorax [3].

### Study groups and outcomes

Among the study population three groups of patients were identified based on the etiology of the ARF: those with pneumonia (Group A), those with acute exacerbation of fibrosis (Group B) and those with other triggers of ARF (Group C). Patients with acute heart failure were not considered in light of the strong evidence recommending the use of NIV in this population [10].

The evaluation of gas exchange was the primary outcome. Clinical failure and length of stay in the hospital (LOS) were secondary clinical outcomes. Clinical failure was defined as the occurrence of any among: in-hospital mortality, ETI and ECMO. In-hospital mortality was defined as death by any cause occurring during hospitalization. LOS was calculated as the number of days from the date of admission to the date of discharge.

### Statistical analysis

All data were analyzed using SPSS (version 18.0) for Mac. Descriptive statistics were reported at baseline, with continuous data expressed as a median (25–75 interquartile range -IQR) and categorical data expressed as counts. Patient characteristics were compared between groups: differences of continuous data between two groups were evaluated by Mann–Whitney test. Differences of categorical variables between two groups were analyzed using the X^2^ test or Fisher exact test where appropriate. A *p* value <0.05 was considered statistically significant.

## Results

### Study population

A total of 60 consecutive patients with DILD undergoing NIV because of an episode of ARF were enrolled during the study period: 63% were males and median (IQR) age was 71 (64–76) years. Demographics, severity of disease, clinical, laboratory and radiological findings on admission before NIV treatment are summarized in Table 1. A total of 28 patients (47%) had a radiological pattern consistent with UIP, 26 patients (44%) had NSIP and 6 (9%) other radiological patterns, including consolidations and ground glass. Clinically, a diagnosis of idiopathic pulmonary fibrosis (IPF) was present in 28 patients (47%), NSIP associated to connective tissue diseases (CTD) in 8 patients, cryptogenic organizing pneumonia (COP) in 3 patients, hypersensitivity pneumonitis (HP) in 1 patients, idiopathic NSIP in 16 patients, hemorrhagic alveolitis in 1 patient and other diagnoses in 3 patients. A first diagnosis of DILD was made in two patients without a previous history of respiratory diseases.

**Table 1:** **Demographics, severity of disease, clinical, laboratory and radiological findings of the study population before non-invasive ventilation**

Characteristic n. (%)	Study population 60 (100)
n. (%)	60 (100)
**Demographics, n. (%)**	
Male	38 (63)
Age, median (IQR) years	71 (64 – 76)
**Comorbidities, n. (%)**	
Chronic Heart Failure	41 (68)
Immunosuppression	39 (65)
Long-term corticosteroid	34 (57)
Immuosuppressive therapy	1 (1.7)
Active cancer	4 (6.7)
Diabetes mellitus	11 (18)
Chronic renal failure	5 (8.3)
Long-Term Oxygen Therapy	29 (49)
**Physical findings on admission, median (IQR)**	
Systolic blood pressure, mmHg	120 (110–139)
Diastolic blood pressure, mmHg	70 (61–80)
Heart Rate, beats/minute	100 (86–111)
Respiratory rate, breaths/minute	30 (27–35)
Oxygen saturation, %	86 (73–94)
**Laboratory values on admission, median (IQR)**	
Arterial pH	7.44 (7.40–7.48)
PaCO_2_, mmHg	40 (35–52)
PaO_2_, mmHg	49 (39–67)
PaO_2_/FiO_2_ ratio	125 (89–167)
White blood cells, cell/L-1	12020 (8390–15400)

### Etiology of ARF

Insufficient clinical data were available for the review committee to characterize the etiology of ARF in 3 patients. A definitive etiology of acute respiratory failure was established by agreement of the review committee in 57 patients. The most frequent causes of ARF in patients with DILD were pneumonia (26 patients, 42%) and acute exacerbation of fibrosis (24 patients, 39%) followed by pulmonary thromboembolism (2 patients, 3.2%), pneumothorax (1 patient; 1.6%), pulmonary neoplasm (1 patient, 1.6%) and other causes (3 patients; 5%). A total of 26 patients (50%) belonged to Group A, 24 patients (39%) to Group B and 7 patients (11%) to Group C.

### Gas exchange during NIV treatment of the study population

A total of 40 patients (67%) were treated with CPAP, and 20 patients (33%) with PSV, see Table 2. The median (IQR) time of NIV treatment among the study population was 72 (28–121) hours. A total of 11 patients (18%) developed intolerance to either the ventilation or the interface during NIV treatment. Neither pharmacological sedation nor physical constraints were used in intolerant patients.

**Table 2:** **Non-invasive ventilation treatment among the study population, including non-invasive pressure support ventilation (PSV) and high-flow non-invasive continuous positive airway pressure (CPAP)**

Characteristic	Study population 60 (100)
n. (%)	60 (100)
**CPAP Treatment**	40 (67)
**CPAP Generator**	
High-flow Venturi	23 (38)
Ventilator	15 (25)
Boussignac Mask	2 (3.3)
**CPAP interface**	
Helmet	28 (70)
Nasal Mask	6 (15)
Face mask	6 (15)
**CPAP Initial PEEP, median (IQR)**	8 (8–10)
**CPAP Initial FiO2, median (IQR)**	50 (50–100)
**PSV Treatment**	20 (33)
**PSV interface**	
Face mask	15 (75)
Nasal Mask	3 (15)
Helmet	2 (10)
**PSV Initial PEEP, median (IQR)**	5 (5–8)
**PSV Initial PS, median (IQR)**	15 (10–20)
**PSV Initial FiO2, median (IQR)**	50 (29–60)

n: number. PEEP: positive end expiratory pressure; FiO2: fraction of inspired oxygen; PS: pressure support; IQR: interquartile range.

**Figure 1:**
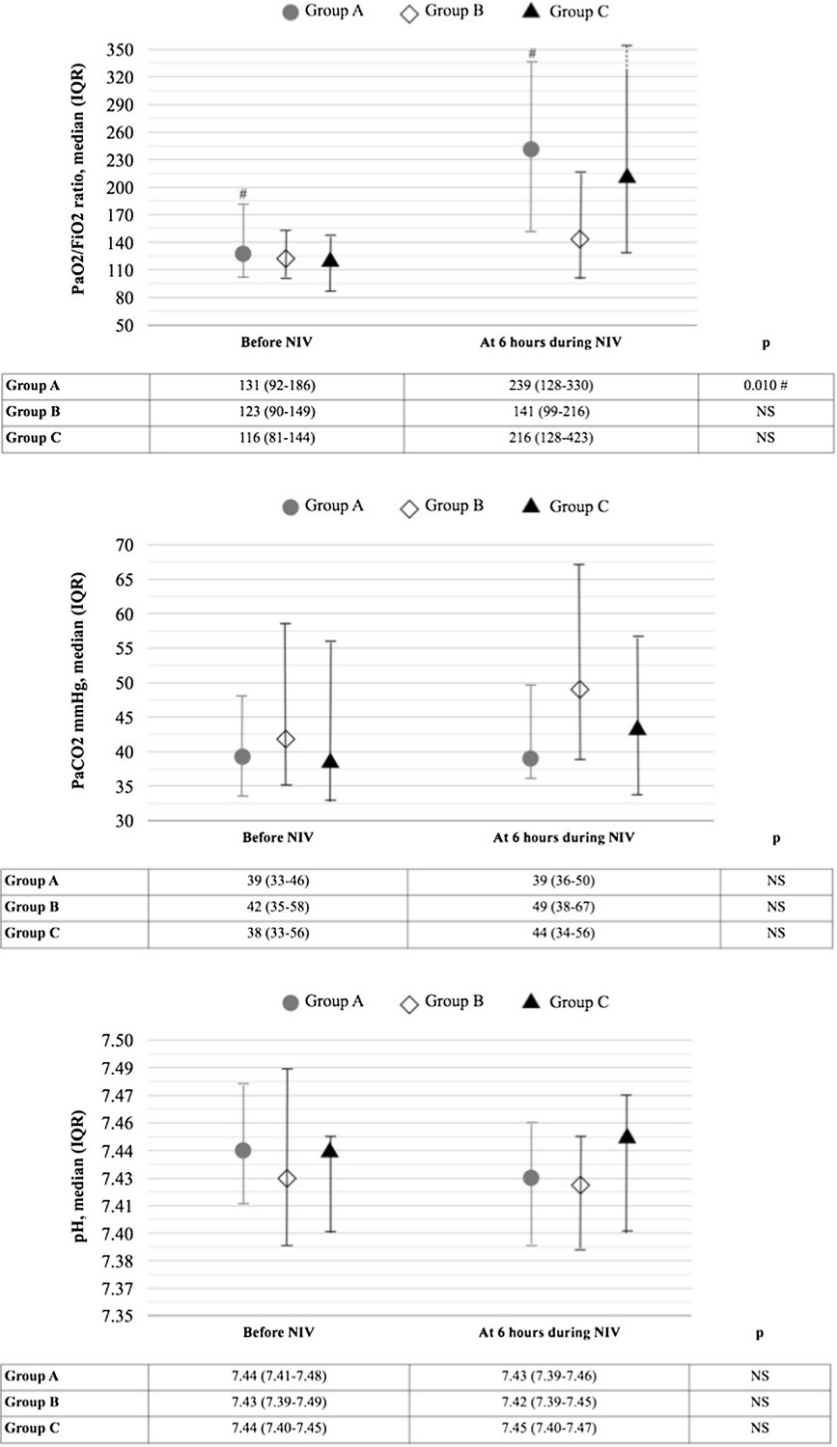
**Gas exchange (PaO**_**2**_ **/FiO**_**2**_ **ratio and PaCO**_**2**_ **) and pH value in the arterial blood before and during non-invasive ventilation (NIV) treatment in the entire study population, in patients whose acute respiratory failure (ARF) was triggered by pneumonia (Group A), by an exacerbation of fibrosis (Group B) and by other causes (Group C).** PaO_2_: partial pressure of oxygen; FiO_2_: Inspired fraction of oxygen; PaCO_2_: partial pressure of carbon dioxide; p = Mann-Whithey test between groups, NS: not significative. No significant difference was detected among the three study groups regarding PaO_2_/FiO_2_ ratio value on admission.

At six hours of NIV treatment, a significant increase in median PaO_2_/FiO_2_ ratio values was detected in the entire study population in comparison to baseline (189 vs. 125, p = 0.001). A significant increase of PaO_2_/FiO_2_ ratio at six hours during NIV treatment was detected in patients whose ARF was triggered by pneumonia (Group A, p = 0.010), but not among those whose ARF was triggered by an acute exacerbation of fibrosis (Group B), see Figure 1. No significant differences at six hours during NIV treatment were detected in PaCO_2_ or pH values in comparison to baseline in the entire population or in the two study groups.

No significant difference was detected in PaO_2_/FiO_2_ ratio, PaCO_2_ or pH values at six hours during NIV treatment in comparison to baseline in patients whose fibrosis had a radiological pattern of UIP or NSIP.

### Clinical outcomes of study population

The median (IQR) LOS in the entire study population was 18 (12–29) days. A clinical failure was detected in 22 patients (37%). Among those, 3 patients (4.6%) were treated with ECMO, 11 patients (18%) were intubated and 21 patients (35%) died during hospitalization, see Table 3. Clinical failure occurred in 10 patients (39%) whose ARF was triggered by pneumonia (Group A) and in 11 patients (46%) whose ARF was triggered by acute exacerbation of fibrosis (Group B), p = 0.775. No significant differences in any of the clinical outcomes evaluated during hospitalization were detected between the two study groups. No significant differences in clinical failure were detected among patients whose fibrosis had a radiological pattern of UIP vs. NSIP. At the univariate analysis the only factors significantly associated with clinical failure were low systolic blood pressure and high respiratory rate before NIV treatment, see Table 4.

**Table 3:** **Clinical outcomes of the study population during hospitalization, according to the three study groups**

	Entire study population (N = 60)	Group A (N = 26)	Group B (N = 24)	Group C (N = 7)	P*
**Clinical failure**	22 (37)	10 (39)	11 (46)	1 (14)	0.320
Endotracheal intubation	11 (18)	4 (15)	6 (25)	1 (14)	0.647
ECMO	3 (4.6)	1 (3.8)	2 (8.4)	0	0.789
In-hospital mortality	21 (35)	9 (35)	11 (46)	1 (14)	0.298

P* = among three group; ECMO: extra-corporeal membrane oxygenation; Group A: patients with either pneumonia or acute heart failure as trigger of acute respiratory failure (ARF): Group B: patients with acute exacerbation of fibrosis as trigger of ARF; Group C: patients with other triggers of ARF.

**Table 4:** **Demographics, severity of disease, clinical, laboratory and radiological findings of the study population according to the presence of a clinical failure during hospitalization (any among: endotracheal intubation, treatment with extra-corporeal membrane oxygenation and in-hospital mortality)**

Characteristic	Absence of clinical failure	Presence of clinical failure	p
n. (%)	38 (100)	22 (100)	
**Demographics, n. (%)**			
Male	22 (58)	16 (77)	0.251
Age, median (IQR) years	71 (64–76)	70 (65–77)	0.842
**Comorbidities, n. (%)**			
Chronic Heart Failure	27 (71)	14 (64)	0.552
Immunosuppression*	23 (60)	16 (73)	0.167
Diabetes mellitus	8 (21)	3 (14)	0.474
Chronic renal failure	4 (11)	1 (4.5)	0.389
Long-Term Oxygen Therapy	22 (58)	7 (33)	0.071
**Physical findings on admission, median (IQR)**			
Systolic blood pressure, mmHg	130 (114–141)	120 (104–130)	0.037
Diastolic blood pressure, mmHg	70 (70–80)	70 (60–80)	0.342
Heart Rate, beats/minute	100 (82–112)	102 (90–111)	0.580
Respiratory rate, breaths/minute	30 (26–32)	35 (27–42)	0.023
Respiratory rate > 30breaths/minute, n.(%)	9 (26)	12 (67)	0.004
Oxygen saturation, %	89 (77–94)	85 (73–90)	0.304
**Laboratory values on admission, median (IQR)**			
Arterial pH	7.43 (7.40-7.46)	7.45 (7.41-7.48)	0.255
Arterial pH < 7.35, n. (%)	4 (11)	2 (9.1)	0.616
PaCO_2_, mmHg	40 (36–55)	38 (33–43)	0.163
PaO_2_, mmHg	49 (40–65)	49 (38–72)	0.945
PaO_2_/FiO_2_ ratio	131 (95–184)	104 (84–135)	0.078
White blood cells, cell/L-1	11820 (7975–15140)	13860 (8515–15632)	0.363
**Radiological appearance of pulmonary fibrosis, n. (%)**			
Usual Interstitial Pneumonia	15 (40)	13 (59)	0.142
Non-Specific Interstitial Pneumonia	8 (21)	6 (27)	0.583

*Immunosuppression defined as the presence of any among: long term corticosteroid, immunosuppressive therapy, active cancer.

## Discussion

This study shows that in patients with DILD undergoing an episode of ARF the improvement in gas exchange during NIV treatment depends on the etiology of the ARF, but not the radiological pattern of DILD. Particularly, an improvement in oxygenation during NIV is detected when pneumonia, but exacerbation of fibrosis, is the triggers of ARF. The occurrence of a clinical failure can be identified in 37% of DILD patients undergoing NIV, with no differences between UIP and NSIP pattern or between ARF etiologies.

So far no randomized controlled trial has evaluated the efficacy of NIV in patients with DILD undergoing an episode of ARF. Although a lack of data exists on this topic, NIV is often used in DILD patients during an acute deterioration in daily clinical practice. Furthermore, NIV use in this population of patients is increasingly taken into account in light of several data showing high rates of mortality in patients with DILD undergoing ETI in the ICU [11–13]. In light of the heterogeneity in terms of both fibrosis and etiologies of ARF, the identification of the right patient to which propose a trial of NIV is crucial in the management of patients with DILD and ARF. Our study did not identify differences in NIV response in terms of oxygenation based on the type of DILD, but the cause of ARF. Particularly, we indirectly confirm the efficacy of NIV in terms of alveolar recruitment during episodes of pneumonia in light of the improved PaO_2_/FiO_2_ ratio during ventilation compared to spontaneous breathing. Cosentini et al. demonstrated that the application of non-invasive CPAP improves oxygenation in patients with pneumonia and reduces the risk of meeting ETI criteria in patients with severe hypoxemic ARF due to pneumonia compared to oxygen therapy [14, 15]. More recently, Carrillo et al. demonstrated that outcome of NIV applied to patients with severe CAP is significantly better in patients with chronic cardio-pulmonary diseases than in *denovo* ARF [16]. On the other hand, the use of NIV in patients with acute exacerbation of fibrosis does not seem to guarantee an improvement in oxygenation in our cohort of patients, exposing them to possible NIV-related adverse events, such as pneumothorax or pneumomediastinum. We recently reported evidence of morphological and physiological effects of the application of non-invasive CPAP during ARF sustained by pneumonia in a patient who underwent lung transplantation because of IPF [17]. We found that the application of CPAP equally increased lung volumes of two hemithoraces affected by different diseases, IPF and pneumonia, evaluated by thorax CT scan. The application of a positive pressure on the IPF lung resulted in minimal overdistention, while in the transplanted lung with pneumonia we detected a recruitment of consolidated areas.

Our data did not allow identification of an advantage in terms of clinical outcomes of NIV in DILD patients. The high rate of negative outcomes recorded in our population is in accordance with previous data and it should be interpreted in the light of the severity of the underlying disease and of the severity of ARF. However, DILD patients represent a population suffering a high mortality and therapeutic options in these subjects are very limited. NIV might also be an alternative option in DILD patients to relieve dyspnoea, in line with the recently published data by Nava and coworkers on patients with end-stage cancer [18].

Our findings have important implications from both a clinical and a research point of view. In clinical practice, NIV could be considered an option in patients with DILD whit an ARF triggered by pneumonia to improve oxygenation. How this physiological benefit could be translated in a better clinical outcome need to be demonstrated in a controlled perspective randomized trial. A special indication for NIV in DILD might be in those patients who have an indication for lung transplant, although no data have been published on this topic. NIV is not a contraindication for lung transplantation as prolonged invasive ventilation is. Moreover, NIV may theoretically work as a “bridge” for patients on waiting list for lung transplantation as it has been shown for other chronic rapid-evolving lung diseases, such as cystic fibrosis [19]. From a research point of view, our data could be of help in designing further prospective observational or interventional studies to demonstrate the effectiveness of NIV, in adjunct to standard medical treatment, in homogeneous clinical-radiological patterns of DILD.

Our study has several limitations. First, data were retrospectively collected which may have led to potential bias in the evaluation and characterization of the etiology of ARF among centers as well as the collection of information on do-not-intubate orders. However, a committee of experts in both DILD and NIV reviewed each single case in order to decrease this risk. In the same way, it should be acknowledged that, although all the centers share a long-term experience with both NIV and non-invasive CPAP, heterogeneity exists in the way CPAP/NIV were administered which might have had an impact on patients’ outcomes. Second, although this was a multicenter study, the limited number of patients did not allow us to highlight possible differences between PSV and CPAP, and led us to use a combined clinical outcome. However, every single component of the combined outcome is recognized as clinical failure for NIV. Finally, ours is a heterogeneous population of DILD patients; this is mainly due to the fact that DILD are rare and NIV is still an emerging treatment in these patients. In light of this, we should also acknowledge as limitation of our study the fact that we were not able to calculate an exact sample size of the study population. The strength of this study is the evaluation of consecutively enrolled patients in six Italian HDUs with long experience in the NIV use [20]. Furthermore, this is the largest cohort of DILD patients specifically treated with NIV, outside the ICU.

## Conclusions

Our results suggest that NIV treatment should be individualized in patients with DILD undergoing an episode of ARF according to the etiology, but the radiological pattern. A trial of NIV may be considered in patients with ARF caused by pneumonia in order to improve gas exchange, although no favorable impact on clinical outcomes has been proven so far. Larger controlled studies are needed to confirm these preliminary findings.

## Abbreviations

ARF: Acute respiratory failure
COP: Cryptogenic organizing pneumonia
CPAP: Continuous positive airway pressure
CTD: Connective tissue diseases
ECMO: Extra-corporeal membrane oxygenation
ETI: Endotracheal intubation
HDU: High dependency units
HP: Hypersensitivity pneumonitis
HRCT: High resolution CT
ICU: Intensive care unit
DILD: Diffuse interstitial lung diseases
IPF: Idiopathic pulmonary fibrosis
IQR: Interquartile range
LOS: Length of stay in the hospital
NIV: Noninvasive ventilation
NSIP: Non-specific interstitial pneumonia
PSV: Pressure support ventilation
RCT: Randomized clinical trial
UIP: Usual interstitial pneumonia.
